# Selecting Risk of Bias Tools for Observational Studies for a Systematic Review of Anthropometric Measurements and Dental Caries among Children

**DOI:** 10.3390/ijerph18168623

**Published:** 2021-08-15

**Authors:** Rokiah Mamikutty, Ameera Syafiqah Aly, Jamaludin Marhazlinda

**Affiliations:** 1 Department of Community Oral Health and Clinical Prevention, Faculty of Dentistry, University of Malaya, Kuala Lumpur 50603, Malaysia; rokiah73@gmail.com (R.M.); drfasya@gmail.com (A.S.A.); 2 Oral Health Programme, Ministry of Health Malaysia, Federal Government Administrative Centre, Putrajaya 62590, Malaysia

**Keywords:** child, systematic review, methods, observational study, bias

## Abstract

In conducting a systematic review, assessing the risk of bias of the included studies is a vital step; thus, choosing the most pertinent risk of bias (ROB) tools is crucial. This paper determined the most appropriate ROB tools for assessing observational studies in a systematic review assessing the association between anthropometric measurements and dental caries among children. First, we determined the ROB tools used in previous reviews on a similar topic. Subsequently, we reviewed articles on ROB tools to identify the most recommended ROB tools for observational studies. Of the twelve ROB tools identified from the previous steps, three ROB tools that best fit the eight criteria of a good ROB tool were the Newcastle–Ottawa Scale (NOS) for cohort and case-control studies, and Agency for Healthcare Research and Quality (AHRQ) and the Effective Public Health Practice Project (EPHPP) for a cross-sectional study. We further assessed the inter-rater reliability for all three tools by analysing the percentage agreement, inter-class correlation coefficient (ICC) and kappa score. The overall percentage agreements and reliability scores of these tools ranged from good to excellent. Two ROB tools for the cross-sectional study were further evaluated qualitatively against nine of a tool’s advantages and disadvantages. Finally, the AHRQ and NOS were selected as the most appropriate ROB tool to assess cross-sectional and cohort studies in the present review.

## 1. Introduction

Assessment of the risk of bias (ROB) or the methodological quality of a study is an essential process in a systematic review and meta-analysis. As recommended by the Cochrane Collaboration, the tools that evaluate the risk of bias assess internal validity, i.e., bias due to flaws in the design, conduct, or analysis of a study that affect its results [1]. Thus, the ROB tools focus on assessing six domains of bias, i.e., selection bias, performance bias, detection bias, attrition bias, reporting bias, and other study biases [2]. Domains unrelated to the ROB or missing the key domains can lead to inaccurate assessments of the ROB. It is important to note that the ROB assessment differs from the overall quality assessment of a study, which refers to assessing internal and external validity, quality of reporting and best research practices, e.g., ethical approval [1,3].

Assessing the ROB of the included studies in a systematic review is critical for several reasons. First, to reduce the tendency to overestimate the treatment effect by having flawed methodological quality studies in meta-analysis [1]. Second, to assist in defining the strength of evidence in Grading of Recommendations, Assessment, Development and Evaluations (GRADE) analysis. Finally, to explore the difference in summary; the effect measures based on the studies’ methodological quality using sensitivity analysis [1]. Furthermore, the ROB of included studies can over or underestimate the outcome effects due to study design, conduct, or analysis of the study [3].

Therefore, it is vital to select the most appropriate ROB tool for a specific review. In order to achieve that, it is essential to comprehend the review topic and previous reviews on a similar topic [4]. For instance, for our present systematic review on the association between anthropometric measurements and dental caries, the selected ROB tools must suit the study design of the included primary studies [5]. Identifying ROB tools employed by other researchers reviewing a similar topic may also provide some insight into the appropriate ROB tools and expected study design for the proposed review [4].

The present systematic review’s topic required ROB tools for observational studies. However, there is little consensus on the best ROB tools for observational studies other than the ROB tools for randomised control trials [2,5]. The Cochrane Collaboration tools for randomised control trials are well-established, validated, reliable and widely use and readily available for researchers who perform clinical systematic reviews [1]. In contrast, although many ROB tools were developed for observational studies, consensus on the best approach to assess the risk of bias in observational studies is inadequate [6]. The existing ROB tools for observational studies differ in their content, reliability, and validity [1,7,8]. Hence, choosing the most appropriate tools for assessing the ROB for observational studies is not easy [6,7]. In the absence of a gold standard for ROB tools for observational studies, identifying the most common tools recommended or cited for use would be useful and very valuable [4,9,10].

Eight criteria to choose an appropriate tool for ROB assessment for a systematic review of observational studies were suggested [1,3,4,5,7,8]. First, the ROB tool must be a simple checklist rather than a scale [8]. Second, the ROB tool should be specific to the study designs and topics under review [5,8]. Third, it possesses a lesser number of key domains [8]. Fourth, the ROB tool should report each domain’s ratings rather than an overall score [1,3]. Fifth, each item should have clear definitions and be transparent regarding each domain’s empirical or theoretical basis [3]. Sixth, the tools chosen should concentrate on assessing the sources of bias [8]. A recent article suggested that ROB tools for observational studies should include questions addressing nine domains, i.e., selection, exposure, outcome assessment, confounding, loss of follow-up, analysis, selective reporting, conflicts of interest and other biases [3]. Seventh, the tool should be rigorously and independently tested for usability, validity, and reliability [1,3,8]. Finally, the ROB tools should be appropriate for the undertaken tasks, for instance, the duration taken to complete each instrument and its ease of use and understanding [4,5].

Another important aspect in selecting appropriate ROB tools is the independent testing for inter-rater reliability of the selected tools among the reviewers and usability of the tool for the review topic [3]. The usability can be measured by ease of use and the time taken to complete the task [4,6,10]. Thus, this study aimed to systematically assess and determine the most appropriate ROB tools of observational studies, assess the inter-rater reliability of the selected ROB instruments, and summarised qualitative pros and cons regarding the usability of each instrument. In the absence of a single prominent tool for the ROB of observational studies, the findings would help others decide which ROB tools to use to assess study quality in systematic reviews of observational studies.

## 2. Materials and Methods

This study was performed within a systematic review and meta-analysis, exploring the association between anthropometric measurements and dental caries among children in Asia to select the most appropriate ROB tool for observational studies (International prospective register of systematic review PROSPERO ID: CRD42019120547). This study employed several steps to select the final ROB tool, an adapted approach by Hootman et al. [4]. The steps include selecting instruments, assessing the inter-rater reliability of the selected instruments, and qualitatively assessing each tool’s pros and cons and the appropriateness of the tools for the review task.

### 2.1. Selecting the Most Appropriate ROB Tools for Observational Studies in a Review of Anthropometric Measurements and Dental Caries among Children (Selecting the ROB Instruments)

#### 2.1.1. Identifying the ROB Tools Used in the Previous Reviews of a Similar Topic

In this step, the most common ROB tools used in previous systematic reviews of anthropometric measurements and dental caries were examined. First, the search for systematic reviews and meta-analyses related to anthropometric measurements and dental caries was performed using six databases: Medline, PubMed, Web of Science, Scopus, CINAHL, and Google Scholar [11]. The search was executed from inception to 30th June 2020 using adapted search strategies validated by the information specialist for the present review on Anthropometric Measurements and Dental Caries in Children in Asia (see supplementary materials, Table S1).

After deduplication of the retrieved reviews, eligibility criteria (see supplementary materials, Table S2) were applied to the title/abstract screening, followed by the full-text screening on the remaining studies by two calibrated reviewers (R.M. and A.S.A.) (title and abstract screening, κ = 0.96, *p* < 0.05; full-text screening, κ = 0.85, *p* < 0.05). Next, the first reviewer (R.M.) extracted the data, including the authors’ name, year of publication, the objective of the reviews, ROB tools used, and the study design of the included studies. The second reviewer (A.S.A.) then verified the extracted data, and discrepancies were resolved by consensus.

#### 2.1.2. Identifying the Most Recommended ROB Tools for Observational Studies

Using PubMed and Google scholar databases, eleven articles regarding ROB tools for observational studies were identified and evaluated. Data on the most recommended ROB tools suggested by the articles [2,3,5,7,8,9,12,13,14,15] were extracted into a spreadsheet and grouped into two categories, i.e., multi-design ROB tools or design-specific tools. Multi-design tools are designed to assess the methodological quality of more than one study design in a single tool, while design-specific tools are checklists that comprise separate checklists according to specific study design [4,15]. As such, multi-design ROB tools that examine non-randomised studies (NRS), including observational studies such as cohort, case-control, and cross-sectional [1], were selected when recommended [4].

#### 2.1.3. Selecting the Most Appropriate ROB Tools for the Review

Subsequently, the ROB tools used in previous reviews of a similar topic and recommended ROB tools listed in the spreadsheet were examined against eight criteria: simple checklist/scale [8], specificity for study design [5,8], number of key domains [8], rating of the domain/overall score [1,3], clear definition of each item [3], concentration on the source of bias [3,8], tested for validity and reliability [1,3,8], and appropriateness for the task [4,5]. Then, the tools with the best fit for the eight criteria were shortlisted for calibration.

### 2.2. Calibration and Inter-Rater Reliability Test of the Selected ROB Tools

A preliminary search of primary studies on anthropometric measurements and dental caries among children in Asia were performed using 26 predetermined databases from 1 April 2019 until 30 June 2019 to identify primary studies for a calibration exercise. Two reviewers independently screened the retrieved articles following the eligibility criteria at two levels: title and abstract, and full-text screening (see supplementary materials, Table S3). Of the 66 eligible primary studies, 64 were cross-sectional, and two were cohort studies. Seven studies (10%) were selected for calibration [16], i.e., five cross-sectional (randomly selected) and both cohort studies, to assess the inter-rater reliability of each selected ROB tool.

As for the results of the above steps for selecting the instruments, three ROB tools or instruments were shortlisted for calibration. The Newcastle–Ottawa Scale (NOS) was selected for cohort studies, and two ROB tools were selected for cross-sectional studies, namely the Agency for Healthcare Research and Quality (AHRQ), and the Effective Public Health Practice Project (EPHPP). Two ROB tools were selected for cross-sectional studies as there was no single most recommended tool.

#### 2.2.1. Selected ROB Tools (Instruments)

The NOS [17] consists of three domains, namely, selection (4 items), comparability (1 item), and outcome (3 items) (see supplementary materials, Table S4). A checklist and coding manual language specific to the current review topic was prepared. When a primary study meets the methodological expected standard, one star was awarded for each item in selection and outcome domains, and a maximum of two stars were awarded for the comparability domain. Studies with NOS star scores from 0 to 4, 5 to 6, and 7 to 9 were considered as having a high, moderate, and low ROB, respectively [18].

The AHRQ [19] contains 11 items and is rated based on the overall score (see supplementary materials, Table S5). For each item, one score is awarded if the quality of the study meets the methodological standard. A score of 0 to 4 indicates a high ROB, 5 to 7 indicates a moderate ROB, and 8 to 11 indicates a low ROB [20].

The EPHPP assesses the ROB for randomised and non-randomised studies (including cohort, case-control, and cross-sectional studies) [21]. The checklist consists of six domains: selection bias (two items), study design (four items), confounders (two items), blinding (two items), data collection method (two items), and withdrawal/dropout (two items) (see supplementary materials, Table S6). Each domain is rated as either weak (if one or more do not meet the expected standard), moderate (if one of the items rated as likely), or strong (all items meet the expected standard). Then a global rating is determined, either weak (two or more domains rated as weak), moderate (one domain rated as weak), or strong (no weak rating) quality is assigned for each article. A guide is provided to assist the rating.

#### 2.2.2. Rating Procedures

The first reviewer developed and piloted two separate spreadsheets for cohort studies and cross-sectional studies, complete with coding rules and operational definitions for the items in each ROB tool to assist in the assessment. The first reviewer randomly selected five cross-sectional studies and all cohort studies (two studies). R.M. and A.S.A. rated two cohort studies with the NOS tool and five cross-sectional studies with the EPHPP and AHRQ tools. A third rater (M.J.) provided consensus where necessary.

Consensus scores were determined as follows: (i) if rater one and two scored similarly, then this score would be used as consensus; (ii) if rater one and two scored differently, the agreed scores after discussion were used as consensus; (iii) if a consensus was not reached then the third rater provided the consensus score and the final decision was agreed upon by all three raters [1,22].

#### 2.2.3. Data Analysis

Data analysis was performed with descriptive and reliability statistics using SPSS version 23 (IBM Corp. Armonk, NY, USA). The descriptive analysis consists of individual rater scores for each item of each instrument, consensus score, total agreements, and qualitative rating.

For the NOS, the total score is continuous. However, the inter-class correlation coefficient (ICC) could not be generated as there were only two cohort studies; therefore, the overall percentage agreement for 18 items was used to measure inter-rater reliability.

For AHRQ, while each item is a categorical variable, the total score is a continuous variable. Thus, the inter-class correlation coefficient (ICC) was used to measure inter-rater reliability in assessing the total ROB scores of five primary studies. A two-way mixed model was applied as the raters were fixed, and the included primary studies were chosen randomly. Absolute agreement was chosen for the type of analysis as the aim was to achieve an agreement between rater two and rater one.

The ICC was then categorised, and the relationship between two raters was defined as ‘little or none’ if the ICC value was 0.25 or below, ‘fair’ if the ICC value was between 0.26 and 0.50, ‘moderate to good’ if the ICC value was between 0.51 and 0.75, and ‘good to excellent’ if the ICC value was 0.76 or above [23]. The kappa score was employed to measure the inter-rater reliability for each item in AHRQ because these items were categorical variables. The kappa score measures agreement between two raters by considering the possibility of the agreement occurring by chance. Kappa statistics were defined as poor (κ ≤ 0.40), fair to good (κ = 0.41–0.74), and excellent (κ ≥ 0.75) [24].

As the EPHPP scores are categorical (‘yes’, or ‘unclear’ or ‘no’), inter-rater reliability for total rating and domains rating was also assessed with kappa statistics.

### 2.3. Qualitative Evaluation of Pros and Cons of the Selected ROB Tools

**Figure 1:**
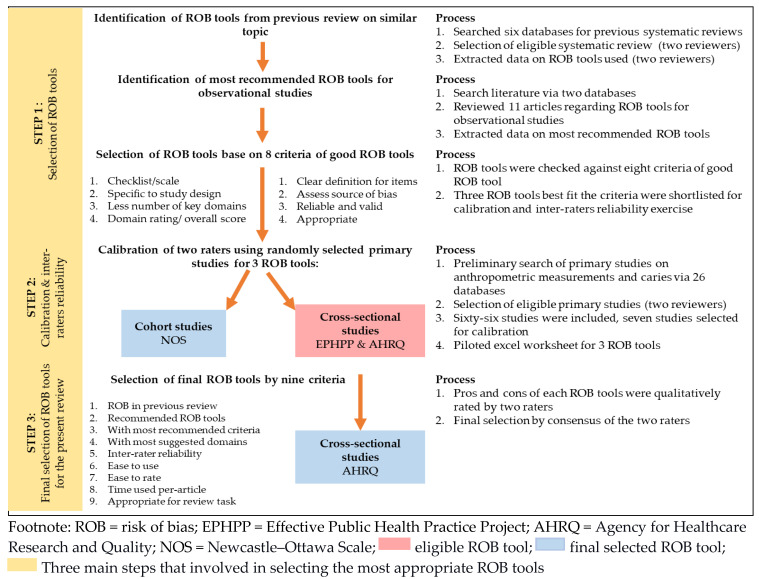
Process of selecting the most appropriate ROB tools for the present review.

As there were two selected ROB instruments for the cross-sectional study, the final selection for the cross-sectional ROB tool was determined by nine criteria evaluating the pros and cons of both ROB tools. Based on the literature review, the nine criteria were (i) the use of the tool in previous reviews [4], (ii) most used/recommended by literature [1,4], (iii) contains the most criteria suggested for ROB tools [1,3,4,5,7,8], (iv) contains the most domains suggested by Wang et al. [3], (v) inter-rater reliability (calibration) [1,3,4,8], (vi) ease of use [4], (vii) ease to rate [4], (viii) average time per article [4,25], and (ix) appropriateness for the review task [4]. The answers were qualitatively discussed among the two raters, and a consensus was reached for all nine items. The process of selecting the most appropriate ROB tools for this review is summarised in Figure 1 below.

## 3. Results

### 3.1. Selection of ROB Instruments

#### 3.1.1. ROB Tools Used in Previous Reviews

This study retrieved twelve systematic reviews and meta-analyses on anthropometric measurements and dental caries among children. The most common study designs were observational studies, including cross-sectional, cohort, and case-control studies, while the most common ROB tool used was Downs and Black. The ROB tool employed, changed from multi-design in earlier reviews to design-specific in the more recent reviews. These reviews used eleven different ROB tools to assess the methodological quality of the included studies, as depicted in Table 1. Of the eleven ROB tools, three tools were not identified by a specific name, four were multi-design tools (Downs and Black, Methodological Evaluation of Observational Research Checklist (MEVORECH), The National Institute of Health (NIH) and Risk of bias in non-randomised studies - of interventions (ROBINS-I)), three were design-specific (Agency for Healthcare Research and Quality (AHRQ), Joanna Briggs Institute (JBI), Appraisal tools for Cross-Sectional Studies (AXIS)), and one tool, i.e., Strengthening the Reporting of Observational studies in Epidemiology (STROBE) was a checklist for reporting observational studies and not an ROB tool. As such, STROBE was omitted from further assessment.

#### 3.1.2. Recommended ROB Tools for Observational Studies

Eleven articles on selecting, guidance, and recommendation of ROB tools for observational studies were identified. The findings of these articles are summarized in Table 2.

There were twelve most recommended or used ROB tools for observational studies as suggested by the eleven articles. These ROB tools were classified into two main groups, multi-design tools and design-specific tools. Similar to previous reviews, the recommended ROB tools changed from multi-design tools to design-specific tools. Of these twelve tools, four were less recommended recently: Zaza, Reisch, Cowley, and Downs and Black. Thus, the remaining eight ROB tools were shortlisted for the next step. Two of the ROB tools were multi-design ROB tools, i.e., EPHPP and Cochrane ROB, while six were design-specific tools, namely, Scottish Intercollegiate Guidelines Network (SIGN), NOS, AHRQ, Critical Appraisal Skills Programme (CASP), Joanna Briggs Institute tools (JBI), and the critical appraisal tool for cross-sectional studies (AXIS). Of these eight ROB tools, four have been used in previous reviews on similar topics (AHRQ, AXIS, JBI, ROBINS-I), and four have not been tested (EPHPP, SIGN, NOS and CASP).

Meanwhile, six tools used in previous reviews (Downs and Black, National Health, Lung, and Blood Institute (NIH), Methodological Evaluation of Observational Research Checklist (MEVORECH), and three other unidentified tools that were not cited as the most recommended tools in the articles were omitted from further assessment [26,27,28].

#### 3.1.3. Most Appropriate ROB Tools for the Included Observational Studies in the Present Review

All eight ROB tools were analysed qualitatively based on eight criteria from the literature, and the findings are presented in Table 3.

Three ROB tools were shortlisted from the findings: NOS for cohort and case-control studies, while AHRQ and EPHPP were shortlisted for cross-sectional studies. NOS was the most used and recommended tool for cohort and case-control studies. Two ROB tools were selected for cross-sectional studies because no single prominent tool was suggested for cross-sectional studies. AHRQ has been cited as the most used for cross-sectional studies in two articles and can be incorporated in RevMan. Whilst, EPHPP has domain rating, is validated and reliable, and includes most of the domains suggested by Wang et al. [3].

Five tools were omitted due to several reasons: i) risk of bias in non-randomized studies of exposures (ROBINS-E) is not fully developed, ii) risk of bias in non-randomised studies of interventions (ROBINS-I) is a tool for non-randomised intervention studies which is not suitable for the task of the proposed review, iii) SIGN and CASP have unclear validity and reliability and were recommended less frequently compared with NOS, iv) JBI is a relatively new ROB tool with no rating and scale, and v) AXIS is a critical appraisal tool with more domains and items but without clear psychometric properties.

### 3.2. Calibration and Inter-Rater Reliability of the Selected ROB Tools

The descriptive calibration findings using NOS for cohort studies and AHRQ and EPHPP for cross-sectional studies are presented in Table 4 and Table 5, respectively.

The NOS consensus scores for both cohort studies were nine, which indicates a low-risk bias. Of the 18 items (nine for each study), both raters agreed with 17 items (Table 4). The total percentage agreement for the NOS scores was 94.4%, indicating excellent agreement between the two raters.

The AHRQ consensus scores for the five cross-sectional studies ranged from 5 to 10 (Table 5). Both raters rated one study as low risk (10 consensus score) and four studies as a moderate ROB (5–7 consensus score). Of 55 items (11 items for each study), a good agreement represented by similar colour boxes between R1 and R2 for items Q1 to Q11 was achieved between both raters for 47 items (85.5%).

The EPPHP consensus rating for the same five studies ranged from moderate to weak quality, i.e., one moderate and four weak quality studies (Table 5). Of the 30 domains (6 domains for each study), 26 domains (86.7%) showed good agreement between the two raters represented by similar colour boxes.

Comparing the AHRQ and EPHPP results, only one study had a similar rating, i.e., Begum et al., rated as moderate (Table 5). The EPHPP identified a moderate ROB for one study and four weak quality studies. In contrast, the AHRQ identified a low ROB for one study and four studies were rated as moderate.

Inter-rater reliability for the AHRQ overall score (ICC = 0.91; 95% CI 0.066 to 0.991; *p* < 0.05) was good to excellent. Inter-rater reliability by items for AHRQ ranged from κ = 0.063 to 1. The lowest scoring items for the AHRQ tool were Q10, confounding (κ = 0.063, *p* > 0.05); Q6, examination method (κ = 0.167, *p* > 0.005); Q7, assessment for quality assurances (κ = 0.375, *p* > 0.05); Q8, and standardised measuring indices (κ = 0.44, *p* < 0.05).

Inter-rater reliability for the EPHPP (κ = 1) showed perfect agreement (global rating) between the two raters. The inter-rater reliability score by domains ranged from κ = 0.167 to 1 and were as follows: selection bias (κ = 1; *p* < 0.05), study design (κ = 1), data collection method (κ = 0.231, *p* > 0.05), and confounder (κ = 0.167; *p* > 0.05).

The summary of the pros, cons, and consensus on the best instrument for cross-sectional studies is depicted in Table 6. According to the review task’s appropriateness, AHRQ was selected because most of the included studies in this review are cross-sectional studies; therefore, the design-specific tools are more appropriate than EPHPP, a multi-design tool.

## 4. Discussion

Assessing the risk of bias of the primary studies included in the systematic reviews and meta-analyses of observational studies is a vital step recommended by the preferred reporting items for systematic reviews and meta-analyses statement [49] and by the meta-analyses of observational studies in epidemiology statement [50]. Meanwhile, selecting an ROB tool for a systematic review examining the health effects of exposure not controlled by investigators (observational studies of exposure) is challenging as there is no consensus on the most recommended ROB tool for observational studies. Therefore, this study aimed to select the most appropriate ROB tools for observational studies in a systematic review of anthropometric measurements and dental caries. After reviewing the ROB tools used in the previous systematic reviews of a similar topic, comparing them with the most used or recommended ROB tools by several articles, and assessing how the tools fit against the eight criteria of good ROB tools, the NOS tool was selected to assess the ROB for cohort studies. The EPHPP and AHRQ tools were selected for cross-sectional studies. Subsequently, after performing the inter-rater reliability and weighing the pros and cons and the appropriateness of the tools specifically for the current review task, AHRQ was selected for cross-sectional studies and NOS for cohort and case-control studies.

There is a dearth of references on the systematic process of selecting the appropriate ROB tools for a systematic review of observational studies. Thus, this study referred to a few articles that assessed the ROB tools in systematic reviews and examined the reliability and validity of selected ROB tools [4,8,15]. Among the approaches used in those articles were: i) reviewing the most used ROB tools in PROSPERO [15], ii) identifying ROB tools through a systematic search via databases and evaluating them for domains related to bias [8], iii) using multiple sources to select the three most used or recommended tools for observational studies, searching for systematic reviews and meta-analyses performed in the field of interest to assess the ROB tools used, then selecting the most mentioned tools in both steps and examining the reliability, validity, and usability of the selected tools [4].

As a result, a systematic search for previous reviews of the same area of interest was performed as the first step. Seven different types of commonly used ROB tools for observational study designs were identified from the twelve reviews examined. Of these seven ROB tools, four were multi-design tools, namely Downs and Black [29,31], NIH [33], ROBINS-I [36], and MEVORECH [32], while three were design-specific tools, namely AHRQ [20], AXIS [35], and JBI [36]. We found that this step was very useful as it provided information on the expected study design of included studies and usability of the selected ROB tools for the current review. As there is no gold standard of ROB tools for observational studies and the ongoing development of new ROB tools, a literature search was performed as the second step to identify the most used or recommended ROB tools for observational studies [4]. From eleven articles on ROB tools for observational studies published between 2002 and 2020, eight tools were used or recommended the most for observational studies, i.e., Cochrane ROB, SIGN, NOS, EPHPP, AHRQ, CASP, JBI, and AXIS. Some earlier tools are less recommended in recent times [15]. For instance, Reisch is not suitable for systematic review purposes [13]. Down and Black is less recommended because it needs considerable epidemiology expertise, is time-consuming to apply, and is hard to use for case-control studies [9,13]. Similarly, Zaza and Cowley are also less used these days [3,9]. This step is also very helpful in guiding the selection process. It provides information about the popular choice among researchers and the relevance and usability of the most used and recommended tools.

Combining the most recommended or used ROB tools with the ones used in the previous reviews, four of the most used or recommended tools were employed in earlier reviews in the same area of interest. Furthermore, the latest review [36] utilised the ROB tool cited by Ma et al. [9], which suggests that the selection of these tools can also be considered as recommendations. The selection of ROB tools for observational studies moves from multi-design tools to design-specific tools in both steps. Similar findings were also reported by Farrah et al. [15]. This step is helpful as it is justified with evidence when the researchers must decide between two good ROB tools; the multi-design and design-specific tool.

Next, eight criteria were used for the selection of good ROB tools. For the cohort and case-control studies, NOS fulfilled the eight criteria the most. NOS is more commonly used compared with SIGN, AHRQ, CASP, and JBI [12]. NOS has fewer items and domains, it rates according to domain rating, was tested for validity and reliability, and can be incorporated in RevMan analysis. In contrast, ROBINS-E is not fully developed for use; ROBINS-I is more suitable for intervention studies and does not suit the present review’s task. SIGN has more items and domains than NOS, no domain rating, and its validity and reliability are unclear, but it assesses five of the nine sources of bias as reported by Wang et al. [3]. Meanwhile, AHRQ, JBI and CASP have no domain rating, unclear reliability, and validity, and even though a guide is provided, they are recommended less frequently than NOS. These findings affirmed that no single standard ROB tool exists for cohort and case-control studies, but the most frequently used is NOS [9,15,51].

For the cross-sectional study, selecting the ROB tools is more challenging as there is no most recommended tool to date. EPHPP tool complied better with the eight criteria than other ROB tools. However, it is a multi-design tool, meaning this single tool assesses the ROB for more than one type of study design [21], thus cross-sectional studies are rated as low quality compared with other study designs. Most included studies are cross-sectional studies that are an appropriate study design to achieve the study objectives, while EPHPP is considered not suitable for the review task.

On the other hand, AHRQ is a design-specific tool for cross-sectional studies. It has been cited twice as the most used tool for cross-sectional studies. The final selection for the ROB tool for cross-sectional studies was performed after calibrating and assessing the tool’s appropriateness for this review task. For several reasons, the AHRQ was more appropriate as the ROB instrument for the present review. Firstly, from the preliminary search, the included studies (66 studies) for the present review are mostly cross-sectional studies (64 studies). Therefore, a tool that is specific for assessing methodological quality for cross-sectional studies is essential. Hootman et al. [4] reported that for a review that included only observational studies, using an instrument with study design-specific criteria may provide the most useful information for assessing quality. Furthermore, AHRQ is recommended due to the researchers’ frequent use and appropriateness for the current review [9,14,20].

Secondly, the findings from the calibration exercises demonstrated that the ROB ratings of the five primary studies varied greatly between AHRQ and EPHPP, although inter-rater reliability for the overall score was good for both raters. Of the five studies, rating with AHRQ resulted in one low ROB study and four moderate ROB studies. In contrast, EPHPP rated one study as moderate quality (moderate ROB) and four studies as weak quality (high ROB). EPHPP includes study design as one of the domains [21]; thus, most of the primary studies selected for testing resulted in a high ROB mainly due to the design and not genuinely due to methodological bias. The body of evidence was later rated using GRADE that included study design as one of the criteria; thus, selecting AHRQ for the ROB assessment at this stage is more appropriate for the specific review.

The rating system of EPHPP is more stringent compared with AHRQ. For instance, for study conduct and confounders, EPHPP rate confounds according to the number of confounders controlled. But for the AHRQ rate, if the study controls the confounders, it disregards the number of confounders; thus, contributing to the difference in rating scores of both tools. These findings agree with other researchers who also found that the rating of studies may differ by the tools used for the assessment of the ROB [4,10,52]. As such, readers should view the interpretation of the ROB assessments between reviews with caution.

Finally, the AHRQ tool was used and tested in a previous review by Chen et al. [20]. Therefore, the usability of this tool for the current review is justified compared with EPHPP. Nonetheless, AHRQ has some limitations compared with EPHPP. The EPHPP reports ratings for each domain, but the AHRQ provides an overall score. EPHPP is more user-friendly than the AHRQ ROB tool and fits six out of nine domains suggested by Wang et al. [3], while AHRQ fits five of these domains. AHRQ is also difficult to use with no manual or guideline [14] compared with EPHPP. On the other hand, being a design-specific tool that lends accuracy, its appropriateness for this specific review, and being commonly used in many reviews of a similar topic that allows comparison, were among the main attributes of AHRQ. Therefore, a customised manual for AHRQ was developed to standardise ratings between the reviewers, while training and calibration were performed to address these issues [9,25]. Furthermore, D Chen, Q Zhi, Y Zhou, Y Tao, L Wu and H Lin [30] used this tool in their review with some modifications for further clarity. Thus, the present review used the AHRQ ROB tool adapted by Chen et al. [20].

Conversely, several reviews on other topics selected the Newcastle–Ottawa Scale (NOS) to assess ROB for cross-sectional studies. However, we did not include this tool for cross-sectional studies because NOS for cross-sectional studies was not listed as the most recommended or used tool in the previous reviews on the same topic. Furthermore the development of the NOS tool was intended for cohort and case-control studies [17]. The NOS for cross-sectional studies was adapted from the NOS for cohort studies [22]. There is no evidence of validation, poor agreement, and lack of comprehensive manuals [53].

This study possesses a few limitations. We conducted the selection of ROB tools to fit the review topic; thus, the selected tool might not be applicable to other reviews. Furthermore, we only included the most recommended ROB tools; therefore, this study might not capture the newly developed tools during the selection process. The findings also revealed that the development of validated ROB tools for observational studies, especially cross-sectional studies, is essential.

Meanwhile, the possible strength of this study includes the systematic and comprehensive approach used during the selection of the most appropriate ROB tool for the review. This study observed ROB tools used in previous reviews of similar topics, selected the tools that best fit the eight criteria of a good ROB tool, conducted calibration and inter-rater reliability exercises, and qualitatively assessed the appropriateness of the tools for this review’s task.

## 5. Conclusions

In conclusion, there are not many validated and reliable tools developed for observational studies of exposure. Hence, searching for the most appropriate tools demanded a systematic strategy. The design-specific ROB tools were selected for the present review, the AHRQ tool for cross-sectional studies and NOS for cohort and case-control studies. The AHRQ was selected for the present review because it is design-specific, mostly used for cross-sectional studies, and was tested for usability by previous reviews. Meanwhile, NOS is the most used tool for case-control and cohort studies.

## Figures and Tables

**Table 1:** List of eleven ROB tools used in previous systematic reviews.

Authors	Titles	Tools	Study Designs	Comments
Kantovitz et al. [26]	Obesity and dental caries: systematic review	Swedish Council on Technology Assessment in Health Care	Cohort Case-control Cross-sectional	ROB tool name not mentioned
Hooley et al. [27]	Body mass index and dental caries in children and adolescents: a systematic review of the literature published 2004 to 2011	Evaluated based on several criteria to assess the quality of methodology, i.e., representative of the sample, control confounder, BMI measure, dental caries measure	Cohort Case-control Cross-sectional	ROB tool name not mentioned.
Hayden et al. [28]	Obesity and dental caries in children: a systematic review and meta-analysis	Appraisal checklists developed by the University of Wales (HEB Wales critical appraisal checklist)	Cohort Case-control Cross-sectional	Multi design tools
Silva et al. [29]	Obesity and dental caries: systematic review	Downs and Black Of 27 items, 18 items selected.	Cohort Case-control Cross-sectional	Multi design tools
Li et al. [30]	Anthropometric Measurements and Dental Caries in Children: A Systematic Review of Longitudinal Studies	STROBE	Case-control Cohort Cross-sectional nested in a birth cohort study	Not ROB tool
Chen et al. [20]	Association between Dental Caries and BMI in Children: A Systematic Review and Meta-Analysis	AHRQ (Modified version)	Cross-sectional	Design specific
Shivakumar et al. [31]	Body Mass Index and Dental Caries: A Systematic Review	Downs and Black Out of 27, 10 items excluded as it applied for intervention studies.	Case-control Cross-sectional Cohort	Multi design tools
Paisi et al. [32]	Body mass index and dental caries in young people: a systematic review	MEVORECH	Case-control Cross-sectional	Multi design tools
Angelopoulou et al. [33]	Early Childhood Caries and Weight Status: A Systematic Review and Meta-Analysis	NIH	Cross-sectional	Multi design tool
Alshiri et al. [34]	Association between Dental Caries and Obesity in Children and Young People: A Narrative Review	Not applicable (Narrative review)	Case-control Cross-sectional Cohort	Not applicable
Alshehri et al. [35]	Association between body mass index and dental caries in the Kingdom of Saudi Arabia: Systematic review	AXIS	Cross-sectional	Design specific
Manohar et al. [36]	Obesity and dental caries in early childhood: A systematic review and meta-analyses	JBI and ROBINS-I	Cross-sectional nested in a cohort Case-Control Cohort	Design specific

BMI: body mass index, ROB: risk of bias, AHRQ: Agency for Healthcare Research and Quality, NOS: Newcastle–Ottawa Scale, STROBE: Strengthening the Reporting of Observational studies in Epidemiology, MEVORECH: Methodological Evaluation of Observational Research Checklist, NIH: The National Institute of Health- quality assessment tool for observational cohort and cross-sectional studies, AXIS: Appraisal tools for Cross-Sectional Studies, JBI: Joanna Briggs Institute, ROBINS-I: Risk of bias in non-randomised studies - of interventions.

**Table 2:** Summary of the most recommended ROB tools for observational studies.

Authors	Most Used/Recommended ROB Tools (Multi-Design)						Most Used/Recommended ROB Tools (Design-Specific)					
	Downs & Black	Zaza	Reisch	Cowley	Cochrane ROB	EPHPP	SIGN	NOS	AHRQ	CASP	JBI	AXIS
West et al. [5]	C CC	C CC					C CC					
Deeks et al. [7]	C CC	C CC	C CC	C CC		C CC CS		C CC				
Maxwell et al. [12]	C CC							C CC				C CC
Sanderson et al. [8]	Lack of single obvious tool for observational studies											
Higgins et al. [2]	C CC	**	**	**				C CC				
Viswanathan et al. [13]			#					C CC				
Zeng et al. [14]							C CC	C CC	CS	C CC		
NICE	C CC CS				NRS	C CC CS		C CC		C CC		
Wang et al. [3]			##	##								
	All the above tools except Reisch and Cowley were listed, but no recommendation was given.											
Farah et al. [15]	C CC CS				NRS	C CC CS		C CC		C CC	C CC	C CC CS
Ma et al. [9]	*	*	*	*	NRS			C CC	CS		CS	
Conclusion	X	X	X	X	√	√	√	√	√	√	√	√

ROB: risk of bias, Cochrane ROB: Cochrane Risk of bias tools, EPHPP: Effective Public Health Practice Project, SIGN: Scottish Intercollegiate Guidelines Network, AHRQ: Agency for Healthcare Research and Quality, NOS: Newcastle–Ottawa Scale, CASP: Critical Appraisal Skills Programme, AXIS: Appraisal tools for Cross-Sectional Studies, JBI: Joanna Briggs Institute, C: cohort, CC: case-control, CS: cross-sectional, NRS: non-randomised studies, * not recommended nowadays [9], ** not recommended [2], # not for systematic review [13], ## not listed for ROB of observational studies for exposure [3], X: not selected, √: selected, 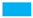 multi-design ROB tools, 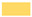 design-specific ROB tools.

**Table 3:** Eight criteria of the recommended ROB tools for observational studies.

No	Criteria	Recommended/Most Used ROB Tools for Observational Studies								
		ROBINS-E	ROBINS-I	EPHPP	SIGN	NOS	AHRQ	CASP	JBI	AXIS
1	Applicability (checklist/ scale)	Risk of bias in non-randomized studies - of exposures (ROBINS-E) (checklist)	Risk of bias in non-randomised studies - of interventions (ROBINS-I) (checklist)	Effective Public Health Practice Project (EPHPP) for quality assessment (checklist)	Scottish Intercollegiate Guidelines Network (SIGN) (Methodology checklist)	Newcastle–Ottawa Scale (NOS) for quality assessment (checklist)	Agency for Healthcare Research and Quality (AHRQ) (Methodology checklist)	Critical Appraisal Skills Programme (CASP) critical appraisal tool (checklist)	Joanna Briggs Institute tools (JBI) for a critical appraisal (checklist)	The critical appraisal tool for Cross-Sectional Studies (AXIS) (checklist)
2	Design specific / multi-design and type of study	Multi-design. Non-randomised studies.	Multi-design. Non-randomised studies of intervention (cohort-like design).	Multi-design. Quantitative studies.	Design-specific. Cohort. Case-control.	Design-specific. Non-randomised studies. Cohort. Case-control.	Design-specific. Cohort. Case-control. Cross-sectional. Case series.	Design-specific. Cohort. Case-control.	Design-specific. Cohort. Case-control. Cross-sectional.	Design-specific. Cross-sectional studies.
3	Number of items and domains	35 Items 7 Domains	34 Items 7 Domains	21 Items 8 Domains	C:18 Items C/C: 15 items 4 Domains	8 Items 3 Domains	C/S: 11 items	C: 12 items C/C: 11 items	8 items	20 items 5 domains
4	Domain rating	Yes	Yes	Yes	No	Yes	No	No	No	No
5	Clear definition of items/ manual	NA	Manual provided	Manual provided	Manual provided	Manual Provided [17]	Hints are Provided	Hints are provided	Manual provided	Manual provided
6	Concentrate on the source of bias (nine domains by Wang et al., [3])	NA	6/9 domains Selection. Exposure. Outcome. Selective reporting. Analysis. Confounders.	6/9 domains Selection. Outcome. Confounding. Loss to follow-up. Analysis.	5/9 domains Selection. Exposure. Confounder. Outcome. Analysis.	4/9 domains Selection. Exposure. Confounder. Outcome.	5/9 domains. Selection. Exposure. Outcome. Confounding. Loss of follow-up.	4/9 domains Selection. Exposure. Outcome. Analys is.	5/9 domains. Selection. Exposure. Outcome. Confounding. Analysis.	5/9 domains Selection. Outcome. Confounding. Analysis. Conflict of interest.
7	Validity and Reliability	N/A	Unclear A Protocol was published to assess the reliability and validity of this tool [25].	Content and construct validity and inter-rater reliability tested [21,37].	Unclear	Established content validity, inter-rater reliability and criterion validity being examined [17].	Unclear Expert consultation.	Unclear Experts piloted checklist [3].	Unclear Peer reviewed.	Unclear Three rounds of the Delphi expert consultation [38].
8	Appropriate for task a. Usability	The tool is under development	For intervention, not exposure. Used in a previous review [36]. Guide to incorporate GRADE [39].	Comprise of global Rating. Easy to use. Good for systematic review [21,37].	Less recommended compared with NOS (Cohort and C/C).	Frequently used Easy to use. May incorporate in RevMan. The best tool for cohort and case-control [14].	Frequently used for CS [14]. Suitable for descriptive cross- sectional studies [9]. Used in a previous review [20]. Can be incorporated in RevMan [40].	Less recommended compared with NOS for cohort and C/C studies.	Preferred for analytic cross- sectional studies and descriptive cross- sectional studies [9]. Used in a previous review [36].	Can be changed and improved where Required. Used in a previous review [35].
	b. Issues/ limitation	This tool is under development. Time-consuming & confusing [6].	For intervention, not exposure. Required substantial epidemiological expertise. Not suitable for present review topic (exposure).			Manual provided. It may be interpreted differently by a different user. Items need to be customised to the review question.	Lack of comprehensive manual which means instruction may interpreted differently by a different user.		No rating/ scale. New tool [9] not much used.	New tools. Critical appraisal tool [9]. Poor inter-rater reliability compared with NOS [41]. No clear psychometrics properties [41].
Shortlisted tools		X	X	√ Multi-design	X	√ Cohort/ Case-control	√ Cross-sectional	X	X	X

ROB: risk of bias, ROBINS-I: Risk of bias in non-randomised studies - of interventions, ROBINS-E: Risk of bias in non-randomized studies - of exposures, EPHPP: Effective Public Health Practice Project, SIGN: Scottish Intercollegiate Guidelines Network, AHRQ: Agency for Healthcare Research and Quality, NOS: Newcastle–Ottawa Scale, CASP: Critical Appraisal Skills Programme, AXIS: Appraisal tools for Cross-Sectional Studies, JBI: Joanna Briggs Institute, NA: not applicable; C/S: cross-sectional studies; C: cohort studies; C/C: case-control; X: ROB tool not selected; **√**: selected ROB tool, 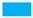 multi-design ROB tools, 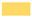 design-specific ROB tools.

**Table 4:** Calibration findings using NOS on two cohort studies.

Article	Rater	Selection					Comparability			Outcome				Total Score	Rating
		Q1	Q2	Q3	Q4	T	Q1a	Q1b	T	Q1	Q2	Q3	T		
Basha et al. [42]	R1	*	*	*	*	4	*	*	2	*	*	*	3	9	Low risk
	R2	*	*	*	*	4	*	*	2	*	*	*	3	9	Low risk
	C	*	*	*	*	4	*	*	2	*	*	*	3	9	Low risk
Li et al. [43]	R1	*	*	*	*	4	*	*	2	*	*	*	3	9	Low risk
	R2	*	*	*	*	4	*	*	2	*	*		2	8	Low risk
	C	*	*	*	*	4	*	*	2	*	*	*	3	9	Low risk

Q: question, T: total stars/score, R: rater; *: star awarded, C: consensus score, 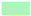 low risk bias.

**Table 5:** Calibration findings using AHRQ and EPHPP ROB tools on five cross-sectional studies.

		AHRQ													EPHPP						
		Items											Total	Rating	Domains						
Article	Rater	Q1	Q2	Q3	Q4	Q5	Q6	Q7	Q8	Q9	Q10	Q11			Selection bias	Study design	Confounder	Blinding	Data Collection method	Withdrawal/ dropout	Global rating
Begum et al. [44]	R1	+	+	+	-	?	+	+	+	NA	-	+	7	M	S	W	M	NA	M	NA	M
	R2	+	+	+	-	?	?	+	+	NA	?	+	6	M	S	W	M	NA	S	NA	M
	**C**	**+**	**+**	**+**	**-**	**?**	**+**	**+**	**+**	**NA**	**-**	**+**	**7**	M	S	W	M	NA	M	NA	M
Diksit et al. [45]	R1	+	+	-	+	?	?	?	+	NA	+	+	6	M	M	W	W	NA	W	NA	W
	R2	+	+	-	+	?	?	?	+	NA	-	+	5	M	M	W	W	NA	W	NA	W
	**C**	**+**	**+**	**-**	**+**	**?**	**?**	**?**	**+**	**NA**	**+**	**+**	**6**	**M**	M	W	W	NA	W	NA	W
Elangovan et al. [46]	R1	+	+	+	+	?	+		+	NA	+	?	7	M	S	W	W	NA	W	NA	W
	R2	+	+	+	+	?	+	+	+	NA	+	?	8	M	S	W	W	NA	S	NA	W
	**C**	**+**	**+**	**+**	**+**	**?**	**+**	**?**	**+**	**NA**	**+**	**?**	**7**	**M**	S	W	W	NA	W	NA	W
Farsi et al. [47]	R1	+	+	+	+	+	+	+	+	NA	+	+	10	L	S	W	W	NA	S	NA	W
	R2	+	+	+	+	+	+	+	+	NA	?	+	9	L	S	W	W	NA	W	NA	W
	**C**	**+**	**+**	**+**	**+**	**+**	**+**	**+**	**+**	**NA**	**+**	**+**	**10**	**L**	S	W	W	NA	S	NA	W
Goodman et al. [48]	R1	+	+	-	+	?	?	?	?	NA	+	+	5	M	S	W	M	NA	W	NA	W
	R2	+	+	-	+	?	+	-	-	NA	+	+	6	M	S	W	W	NA	W	NA	W
	**C**	**+**	**+**	**-**	**+**	**?**	**?**	**?**	**?**	**NA**	**+**	**+**	**5**	**M**	S	W	M	NA	W	NA	W

Q: Question, R1: Rater 1, R2: Rater 2, C: consensus score, +: Yes, ?: Unclear, -: No, NA: Not applicable, M: moderate risk of bias, L: low risk of bias, S: strong quality, M: Moderate quality, W: Weak quality, AHRQ: Agency for Healthcare Research and Quality, EPHPP: Effective Public Health Practice Project

**Table 6:** Pros, cons, and consensus on the best instrument for cross-sectional studies.

Qualitative Characteristic		Descriptions	EPHPP	AHRQ	Consensus
1	Used in previous SR	Tested with a similar review topic	X	√	AHRQ
2	Most used/recommended by literature	Frequently cited by literature	√ (Multi-design)	√ (Design-specific)	Both
3	Contains recommended criteria	1. Methodological checklist	√	√	EPHPP
		2. Reliability and validity	√	Unclear	
		3. Design specific	X	√	
		4. Domain rating	√	X	
		5. Clear definition of items/manual	√	X	
4	Contains most domains as suggested by Wang et al. [3]	a. Selection			EPHPP
		Sample representative of the target population	√	√	
		Comparability of exposure and comparison groups	√	X	
		Appropriateness of eligibility criteria	√	√	
		Recruitment time frame	√	√	
		Non-response rate	√	√	
		b. Exposure			
		Validity and reliability of exposure measurement	√	√	
		c. Outcome assessment			
		Accuracy of outcome measurement	√	√	
		Blinding of the research staff	NA	NA	
		d. Confounding			
		Description of confounding variables	√	√	
		Accounting for confounding	√	√	
		e. Loss of follow-up			
		Adequacy of the length of follow-up	√	√	
		Amount of loss of follow-up	√	√	
		Handling of loss of follow-up	√	√	
		f. Analysis			
		Appropriate statistical method	√	X	
		g. Selective reporting			
		Selective reporting of outcome	X	X	
		h. COI e.g., funding	X	X	
		i. Other bias	X	X	
		Total Domain	6/9	5/9	
5	Inter-rater reliability (calibration)	a. Overall	κ = 1	ICC = 0.91	Both excellent
		b. By item/domain (range)	Κ = 0.167 to 1	Κ = 0.063 to 1	Both need improvement
6	Ease of use		Easy	Difficult	EPHPP
7	Ease of rating values for each item		Easy	Difficult	EPHPP
8	Time per article		30 min	30 min	Both
9	Appropriate for the task	a. Of 66 included studies in the systematic review, 64 were cross-sectional studies, and two were cohort studies; thus, design-specific ROB is more useful.			AHRQ better to rate cross-sectional studies
		b. EPHPP includes study design as one of the domains; thus, it lowered the rating due to study design, making it less suitable.			
		c. AHRQ does not have a clear manual; hence, a manual that is topic-specific should be developed before the actual assessment			

X: No, √: Yes, NA: Not applicable, SR: systematic review, κ: kappa score, ICC: inter-class correlation coefficient, ROB: risk of bias, AHRQ: Agency for Healthcare Research and Quality, EPHPP: Effective Public Health Practice Project, COI: conflict of interest.

## Supplementary Materials

The following are available online at https://www.mdpi.com/article/10.3390/ijerph18168623/s1. Table S1: Search strategy for previous reviews in similar topic. Table S2: Eligibility criteria for selection of previous review on similar topic. Table S3: Eligibility criteria for selection primary studies on anthropometric measurements and dental caries. Table S4: NOS tool. Table S5: AHRQ tool. Table S6: EPHPP tool.
