# Supplementary material for: Selecting Risk of Bias Tools for Observational Studies for a Systematic Review of Anthropometric Measurements and Dental Caries among Children
Source: Int J Environ Res Public Health. 2021 Aug 15;18(16):8623. doi: 10.3390/ijerph18168623 (PMC8391268; doi:10.3390/ijerph18168623)
Supplement: Supplementary file 1 [file ijerph-18-08623-s001.zip › ijerph-1292936-supplementary.pdf]

SUPPLEMENTARY MATERIAL

Table S1: Search strategy for previous reviews in similar topic

	**Search Strategy for PubMed**	**Results**
**#1**	"child"[MeSH Terms] OR "child"[All Fields] OR "children"[All Fields] OR "child s"[All Fields] OR "children s"[All Fields] OR "childrens"[All Fields] OR "childs"[All Fields] OR "adolescences"[All Fields] OR "adolescency"[All Fields] OR "adolescent"[MeSH Terms] OR "adolescent"[All Fields] OR "adolescence"[All Fields] OR "adolescents"[All Fields] OR "adolescent s"[All Fields] OR "toddler"[All Fields] OR "toddler s"[All Fields] OR "toddlers"[All Fields] OR "adolescent"[MeSH Terms] OR "adolescent"[All Fields] OR "teen"[All Fields] OR "adolescent"[MeSH Terms] OR "adolescent"[All Fields] OR "youth"[All Fields] OR "youths"[All Fields] OR "youth s"[All Fields]	3784836
**#2**	"anthropometry"[MeSH Terms] OR "body fat distribution"[MeSH Terms] OR "waist hip ratio"[MeSH Terms] OR "waist height ratio"[MeSH Terms] OR "skinfold thickness"[MeSH Terms] OR "waist circumference"[MeSH Terms] OR "obesity"[MeSH Terms] OR "body mass index"[All Fields] OR ("waist height ratio"[MeSH Terms] OR ("waist height"[All Fields] AND "ratio"[All Fields]) OR "waist height ratio"[All Fields] OR ("waist"[All Fields] AND "height"[All Fields] AND "ratio"[All Fields]) OR "waist height ratio"[All Fields]) OR ("skinfold thickness"[MeSH Terms] OR ("skinfold"[All Fields] AND "thickness"[All Fields]) OR "skinfold thickness"[All Fields]) OR ("waist hip ratio"[MeSH Terms] OR ("waist hip"[All Fields] AND "ratio"[All Fields]) OR "waist hip ratio"[All Fields] OR ("waist"[All Fields] AND "hip"[All Fields] AND "ratio"[All Fields]) OR "waist hip ratio"[All Fields]) OR ("waist circumference"[MeSH Terms] OR ("waist"[All Fields] AND "circumference"[All Fields]) OR "waist circumference"[All Fields]) OR ("anthropometries"[All Fields] OR "anthropometry"[MeSH Terms] OR "anthropometry"[All Fields]) OR ("obeses"[All Fields] OR "obesity"[MeSH Terms] OR "obesity"[All Fields] OR "obese"[All Fields] OR "obesities"[All Fields] OR "obesity s"[All Fields])	871247
**#3**	"caries"[All Fields] OR "dental caries"[MeSH Terms] OR ("dental"[All Fields] AND "caries"[All Fields]) OR "dental caries"[All Fields] OR "caries"[All Fields] OR ("dental caries"[MeSH Terms] OR ("dental"[All Fields] AND "caries"[All Fields]) OR "dental caries"[All Fields]) OR ("dental caries"[MeSH Terms] OR ("dental"[All Fields] AND "caries"[All Fields]) OR "dental caries"[All Fields] OR ("dental"[All Fields] AND "decay"[All Fields]) OR "dental decay"[All Fields]) OR ("dental caries"[MeSH Terms] OR ("dental"[All Fields] AND "caries"[All Fields]) OR "dental caries"[All Fields] OR ("tooth"[All Fields] AND "decay"[All Fields]) OR "tooth decay"[All Fields])	62469
**#4**	Search: **((#1) AND (#2)) AND (#3)** Filters: **Meta-Analysis, Review, Systematic Review**	91

Table S2: Eligibility criteria for selection of previous review on similar topic

No		Inclusion	Exclusion
1	Type of studies	• Review<br>• Systematic review<br>• Systematic review and meta-analysis	• Primary studies
2	Type of population	• age 19-year-old and below	• studies of population restricted to a specific disease, condition, or metabolic disorders.
3	Type of exposure	**Anthropometric measurements include:**<br><br>Body Mass Index (BMI)<br>Waist circumference (WC)<br>Waist-to-hip ratio (WHR)<br>Waist to height ratio (WHtR)<br>Skin fold thickness (SFT)	
4	Type of outcome	**Association between anthropometric measurement and dental caries**<br>(4 types of association: positive, negative, U-shape and no association)<br><br><u>Whereby, dental caries measured by</u>:<br>• **Prevalence** (occurrence of caries or caries status). All variations of grouping were included e.g.<br>• caries-free vs caries group (DMFT=0 & DMFT $\geq$1)<br>• caries-free vs caries group (dmft/deft/dft/=0 & dmft/deft/dft $\geq$1)<br>• **Experience** (number of decays, filled, extracted teeth due to caries).<br>• DMFT/ DFT/DFMS<br>• dmft/dft/deft/dfs<br>• ICDAS	• exclude if not examine association anthropometric and dental caries
5	Limit	English language and published	Non-English and unpublished

Table S3: Eligibility criteria for selection primary studies on anthropometric measurements and dental caries.

No		Inclusion	Exclusion
1	Type of studies	**Observational studies**:<br>• cross sectional<br>• comparative cross-sectional<br>• case-control<br>• nested case-control<br>• retrospective<br>• prospective cohort	• case series<br>• case report<br>• intervention/ experimental study
2	Type of population	• age 19-year-old and below<br>• both genders<br>• in Asian countries	• studies of population restricted to a specific disease, condition, or metabolic disorders.
3	Type of exposure	**Anthropometric measurements**<br><br>**<u>BMI</u>**<br>BMI of any variations were included, e.g. BMI z-score, BMI (CDC), BMI specific for countries and etc<br><br>All 4 groups (underweight, normal weight, overweight, obese) included in the analysis<br><br>If only one group compared with normal weight, e.g., obesity with normal weight, the studies were included if the definition of obesity follows the cut-off point used in the selected BMI.<br><br>**<u>WC/ WHR /WHtR/SFT</u>**<br>Used standard guideline & categorised following the standard guideline<br><br>**Comparator of interest**:<br>BMI: Normal weight/ condition.<br>WC, WHR, WHtR, SFT: Non-obese	• BMI: studies that combined groups for analysis were excluded. e.g., normal weight combined with underweight or overweight with obesity<br>• BMI was measured as average for entire sample group<br><br>• studies used overall mean for WC, WHR, WHtR, SFT<br><br>• studies with another comparator were excluded
4	Type of outcome	**Association between anthropometric measurement and dental caries**<br>(4 types of association: positive, negative, U-shape and no association)<br>Dental caries measured by:<br>• **Prevalence** (occurrence of caries or caries status). All variations of grouping were included e.g.<br>◦ caries-free vs caries group (DMFT=0 & DMFT $\geq$1)<br>◦ caries-free vs caries group (dmft/deft/dft/=0 & dmft/deft/dft $\geq$1)<br>• **experience** (number of decays, filled, extracted teeth due to caries).<br>◦ DMFT/ DFT/DFMS<br>◦ dmft/dft/deft/dfs<br>◦ ICDAS	• exclude if not examine association anthropometric and dental caries<br>• studies examined solely untreated dental caries (dt/ DT) with caries-free were excluded

Table S4: NOS tool for cohort

MANUAL FOR RISK OF BIAS TOOL FOR COHORT STUDY
NEWCASTLE - OTTAWA QUALITY ASSESSMENT SCALE (NOS)

Note: A study can be awarded a maximum of one star for each numbered item within the Selection and Outcome categories. A maximum of two stars can be given for Comparability

Domain	Items and description	Note/ guide
**Selection**<br>(max. 4 star)<br><br>*maximum of one star for each numbered item)	**1. Representativeness of the exposed cohort**<br>a) truly representative of the average _______________ (describe) in the community ✸<br>b) somewhat representative of the average ______________ in the community ✸<br>c) selected group of users e.g. nurses, volunteers<br>d) no description of the derivation of the cohort	Randomly selected e.g. from comprehensive list<br><br>Somewhat likely e.g. referred from clinic in a systematic manner
	**2. Selection of the non-exposed cohort**<br>a) drawn from the same community as the exposed cohort ✸<br>b) drawn from a different source<br>c) no description of the derivation of the non-exposed cohort	
	**3. Ascertainment of exposure**<br>a) secure record (eg surgical records) ✸<br>b) structured interview ✸<br>c) written self-report<br>d) no description	
	**4. Demonstration that outcome of interest was not present at start of study**<br>a) yes ✸<br>b) no	
**Comparability**<br>(max. 2 star)	**1) Comparability of cohorts on the basis of the design or analysis**<br><br>a) study controls for____ (select the most important factor) ✸<br>b) study controls for any additional factor ✸	a. Age, gender, ethnicity<br>b. Diet, OHI, fluoride usage, SES **OR** any others **please specify**.
**Outcome**<br>(max. 3 star)<br><br>*maximum of one star for each numbered item	**1) Assessment of outcome**<br><br>a) independent blind assessment ✸<br>b) record linkage ✸<br>c) self-report<br>d) no description	clinical examination for dental caries by calibrated clinician or secondary data
	**2) Was follow-up long enough for outcomes to occur**<br>a) yes (select an adequate follow up period for outcome of interest) ✸<br>b) no	2 years and more
	**3) Adequacy of follow up of cohorts**<br>a) complete follow up - all subjects accounted for ✸<br>b) subjects lost to follow up unlikely to introduce bias - small number lost, >80 % follow up, or description provided of those lost) ✸<br>c) follow up rate < 80% and no description of those lost<br>d) no statement	Note: For survey research intended to represent all schools and colleges of pharmacy, a response rate of ≥ 80% is expected [1]

Footnotes

[1] J.E. Fincham, Response rates and responsiveness for surveys, standards, and the Journal, Am J Pharm Educ 72(2) (2008) 43-43.

Table S5: AHRQ tool for cross-sectional

No.	Description of question	Criteria
**Q1**	Define the source of information<br>(Survey, record review)	1= from survey<br>2= not mentioned<br>3= records/ unclear info
**Q2**	List inclusion and exclusion criteria for subjects or refer to previous publications	1= clearly mentioned<br>2= no information<br>3= unclear / insufficient info
**Q3**	Indicate whether subjects were consecutive if not population based. Whether subjects are representative of the average in the community?	1= representative<br>2= not representative (convenience/ not randomly selected)<br>3= no clear info
**Q4**	Indicate time period used for identifying subjects	1= time period given<br>2= no info given
**Q5**	Indicate if evaluators of subjective components of study were masked to other aspects of the status of the participants. Are the evaluators professional (trained /calibrated)?	1= evaluator trained/ calibrated<br>2= not calibrated/ trained<br>3= unclear /not mentioned
**Q6**	Is the examination method standard?	1= exposure & o/come method are standard<br>2= not done<br>3= unclear /partially done
**Q7**	Describe any assessments undertaken for quality assurance purposes (e.g., test/retest of primary outcome measurements)	1= exposure & o/come tools validated/examiner-kappa-score reported)<br>2= not done<br>3= unclear /partially done
**Q8**	Are the assessments and classification of caries index and BMI clearly stated and standard?	1= standard classification for both exposure & outcome<br>2= not use standard method<br>3= unclear/ no information
**Q9**	If any, explain any subject exclusions from analysis	1=mentioned clearly<br>2=not mentioned<br>3=unclear information<br>4=NA
**Q10**	Describe how confounding was assessed and/or controlled.	1=mentioned (design/analysis)<br>2=not done<br>3=unclear /not mentioned
**Q11**	Summarize patient response rates and completeness of data collection	1=mentioned & above 80%<br>2=not mentioned<br>3=unclear information

Table S6. EPHPP tool for cross-sectional

Domain	Questions	Rating
**SELECTION BIAS**<br>(2 items)	(Q1) Are the individuals selected to participate in the study likely tobe representative of the target population?<br>1 Very likely<br>2 Somewhat likely<br>3 Not likely<br>4 Can't tell<br>(Q2) What percentage of selected individuals agreed to participate?<br>1 80 -100% agreement<br>2 60 —79% agreement<br>3 less than 60% agreement<br>4 Not applicable<br>5 Can't tell	Strong:<br>Q1=1 & Q2=1<br><br>Moderate:<br>Q1=1/2 & Q2=2<br>OR<br>Q1=1/2 & Q2=5<br><br>Weak:<br>Q1=3/Q2=3 OR<br>Q1=4 &Q2=5
**STUDY DESIGN**<br>(4 items)	Indicate the study design: Cross-sectional<br>3 item not applicable	Weak:<br>Cross-sectional
**CONFOUNDERS**<br>(2 items)	(Q1) Were there important differences between groups prior to the intervention?<br>1 Yes<br>2 No<br>3 Can't tell<br>(02) If yes, indicate the percentage of relevant confounders that were controlled (either in the design (e.g., stratification, matching) or analysis)?<br>1 80 — 100% (most)<br>2 60 —79% (some)<br>3 Less than 60% (few or none)<br>4 Can't Tell	Strong:<br>Q1=2 / Q2=1<br>Q1=1 & Q2=1<br><br>Moderate:<br>Q1=1 & Q2=2<br><br>Weak:<br>Q1=1 & Q2=3/<br>OR<br>Q1=3 & Q2=4
BLINDING<br>(2 items)	Q1) Was (were) the outcome assessor(s) aware of the intervention or exposure status of participants?<br>1 Yes<br>2 No<br>3 Can't tell<br>Q2) Were the study participants aware of the research question?<br>1 Yes<br>2 No<br>3 Can't tell	NA
DATA COLLECTION METHOD<br>(2 items)	Q1) Were data collection tools shown to be valid?<br>1 Yes<br>2 No<br>3 Can't tell<br>Q2) Were data collection tools shown to be reliable?<br>1 Yes<br>2 No<br>3 Can't tell	Strong:<br>Q1=1 & Q2=1<br>Moderate:<br>Q1=2/3 & Q2=1<br>Q1=1 & Q2=2/3<br>Weak:<br>Q1=2/3 & Q2=2/3
WITHDRAWAL/ DROPOUT	Not applicable (cross-sectional)<br>(2 items)	NA
ANALYSIS<br>(4 items)	Q1) Indicate the unit of allocation (circle one): Community OR organization/institution OR practice/office individual<br>Q2) Indicate the unit of analysis (circle one): Community OR organization/institution OR practice/office OR individual<br>Q3) Are the statistical methods appropriate for the study design?<br>1. Yes OR 2. No OR 3. Can't tell<br>(Q4) Is the analysis performed by intervention allocation status (i.e., intention to treat) rather than the actual intervention received?<br>1. Yes OR 2. No OR 3. Can't tell	

Table S6. EPHPP tool (continued)

Component rating

a. Selection bias	Strong	Moderate	Weak
b. Study design	Strong	Moderate	Weak
c. Confounder	Strong	Moderate	Weak
d. Blinding	Strong	Moderate	Weak
e. Data collection method	Strong	Moderate	Weak
f. Withdrawal and dropouts	Strong	Moderate	Weak

Global rating for this paper:

1. Strong (no weak rating)
2. Moderate (one weak rating)
3. Weak (two or more weak rating)

With both reviewers discussing the ratings:

Is there a discrepancy between the two reviewers with respect to the component(A-F) ratings?
1. No 2. Yes

If yes, indicate the reason for the discrepancy

1. Oversight
2. Differences in interpretation of criteria
3. Differences in interpretation of study

Final decision of both reviewers (circle one)

1. Strong
2. Moderate
3. Weak
